# A Conservative Approach to a Large Mycotic Pulmonary Pseudoaneurysm

**DOI:** 10.1155/2021/6456216

**Published:** 2021-11-18

**Authors:** Erin Torpey, Jenna Spears, Yousif Al-Saiegh, Mindi Roeser

**Affiliations:** Department of Medicine, Pennsylvania Hospital, University of Pennsylvania Health System (UPHS), Philadelphia, PA, USA

## Abstract

Pulmonary mycotic pseudoaneurysm is a rare complication of bacteremia with high associated mortality. We present a case of a large proximal pulmonary artery pseudoaneurysm as a result of methicillin-sensitive Staphylococcus aureus bacteremia, originating from a tunneled dialysis catheter infection. This case was ultimately managed conservatively with surveillance imaging and a prolonged intravenous antibiotic course, rather than with surgical or interventional management. To our knowledge, this is the first reported case of a mycotic pulmonary pseudoaneurysm due to septic embolization of an infected superior vena cava thrombus.

## 1. Introduction

A mycotic pulmonary pseudoaneurysm is a rare but deadly consequence of septic emboli in bacterial and fungal bloodstream infections. Timely diagnosis with appropriate imaging is crucial and requires a high index of suspicion. Early detection is important to instate prompt antimicrobial therapy and plan for potential management, as the mortality rate associated with pulmonary pseudoaneurysm rupture is greater than 50% [1].

## 2. Methods

Case report and review of the literature.

## 3. Case

**Figure 1:**
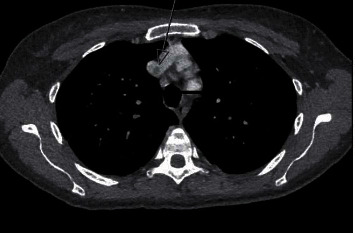
Axial computed tomography angiography depicting a filling defect (arrow) in the upper superior vena cava at the junction of the left and right brachiocephalic veins.

**Figure 2:**
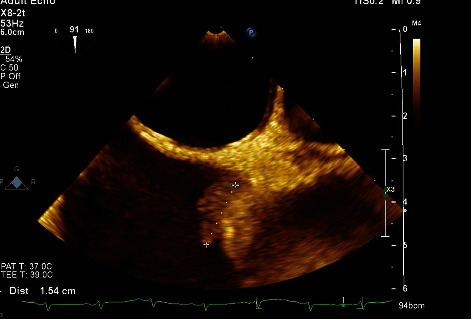
Transesophageal echocardiogram showing a mobile lesion at the junction of the SVC and right atrium. The lesion is irregular and measures about 1.7 cm in greatest dimension.

**Figure 3:**
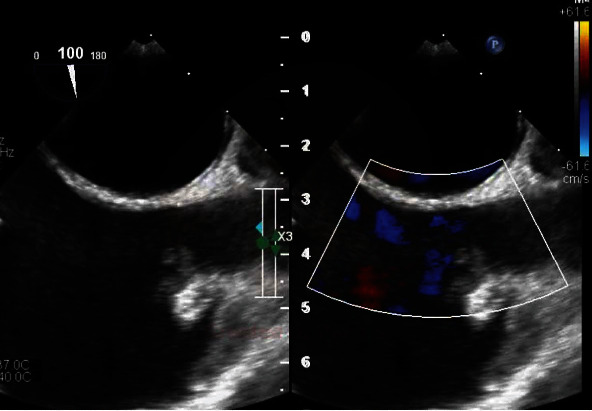
Transesophageal echocardiogram with color Doppler showing mobile thrombus at the junction of the SVC and right atrium.

**Figure 4:**
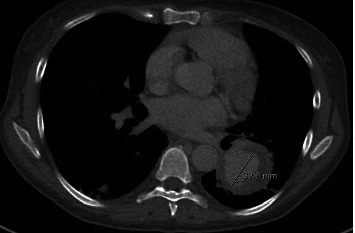
Axial computed tomography angiography depicting a pseudoaneurysm measuring 52 × 47 mm with an irregular central contrast collection measuring 32 mm (marked above).

A 49-year-old female on hemodialysis presented with fever and confusion and was found to have sepsis secondary to a tunneled dialysis catheter (TDC) line infection. The patient was febrile up to 103.1 degrees F, with a pulse of 116 bpm, SpO_2_ was 97% on room air, and blood pressure was 130/71 mmHg. On physical exam, the patient was orientated but slow to respond and globally weak. She was due to have her TDC removed, as she had a newly matured right upper extremity arteriovenous graft. She was empirically started on intravenous vancomycin, cefepime, and metronidazole. Her TDC was removed. She was found to be bacteremic with methicillin-sensitive Staphylococcus aureus (MSSA) and was narrowed to Cefazolin monotherapy. Interestingly, the patient's daily blood cultures remained persistently positive for MSSA for four further days, despite appropriate antibiotics and source removal. The patient was also persistently febrile for the majority of her hospitalization, despite targeted antibiotic therapy. Further investigation of other possible sources of infection ensued, including a computed tomography chest scan with intravenous contrast. This revealed 40-50 bilateral cavitating and noncavitating pulmonary nodules, and a large lower lobe consolidation felt to be secondary to septic emboli. She was also found to have an acute pulmonary embolism (PE) of the distal right segmental pulmonary artery. A filling defect was seen in the upper superior vena cava representing either a thrombus or fibrinous sheath from her previous TDC (Figure 1). Of note, the pulmonary arteries were of normal caliber on this scan. Transesophageal echocardiography was obtained and confirmed the presence of a mobile thrombus at the SVC and right atrial junction measuring 1.7 cm in the greatest dimension (Figures 2 and 3). The patient was subsequently started on a heparin infusion for her acute PE, and vascular surgery was consulted for further evaluation of the mobile thrombus. Vascular surgery felt neither lytic nor surgical intervention was warranted for the SVC thrombus and recommended follow-up imaging to monitor the SVC thrombus in one to two weeks. Ten days after the initial CT scan, a repeat CT chest with IV contrast revealed a new left lower lobe pulmonary artery pseudoaneurysm measuring 52 × 47 mm with irregular central contrast measuring 32 mm in dimension (Figure 4). This defect was felt to be mycotic in nature. Interventional radiology was consulted, who felt the patient was not a candidate for left pulmonary artery embolization due to the risk of left lung infarction. Thoracic surgery was consulted and discussed both conservative and surgical options with the patient. The conservative option included continuing intravenous antibiotics and completing weekly surveillance CT scans, with the hope of spontaneous involution and eventual resolution of the lesion. The second option was a left-sided pneumonectomy, which was deemed high-risk due to the proximity of the pseudoaneurysm to the main pulmonary artery. Ultimately, the patient opted to pursue conservative management and was followed closely by the thoracic surgery and infectious disease team. The patient completed eight weeks of IV Cefazolin. Despite her acute PE, anticoagulation was held in the setting of the large pseudoaneurysm and the associated significant bleeding risk. At a 2-month follow-up, her large pulmonary pseudoaneurysm had decreased in size to 38 × 42 × 40 mm. Notably, the patient had an interval decrease of peripheral thrombus within the pseudoaneurysm and resolution of her chronic right upper lobe pulmonary embolus. The nonocclusive SVC thrombus was found to be unchanged. The patient remained asymptomatic and clinically stable and is now off antibiotics. Given the stability of her pseudoaneurysm, her surveillance CT scans have been spaced out to every six months.

## 4. Discussion

Infection can cause focal aneurysmal degeneration of the arterial wall, leading to either a true or pseudoaneurysm [2]. When bacteremia or septic emboli are felt to be the source of the aneurysm, as was the case in this patient, the lesion is termed a mycotic aneurysm. Mycotic aneurysms are uncommon and most frequently involve the aorta and the cerebral, peripheral, and visceral arteries [2]. Mycotic aneurysms commonly present with symptoms such as fever, chills, back pain, and abdominal pain [3, 4]. There are only several reported cases of mycotic pulmonary artery aneurysms [2]. Although these cases are rare, they carry high associated mortalities of up to 50% [1]. The high mortality may be related to the architecture of the pulmonary arteries, as they lack an adventitial wall layer and are more likely to rupture than systemic aneurysms [1]. Mycotic aneurysms often arise as a result of septic embolization from infective endocarditis [2]. This patient had an atypical source of septic embolization as there was no evidence of endocarditis on transesophageal echocardiography; however, she did have a large thrombus in the distal SVC from an infected TDC, which was felt to be the source. The presentation of an infected aneurysm is dependent on its location [2]. While peripheral lesions may be pulsatile and painful, the involvement of deeper sites may only be associated with fever of unknown origin, as with this patient [2]. Patients with pulmonary artery aneurysms are often otherwise asymptomatic but can present with sepsis, life-threatening hemoptysis, and hypoxemia [2]. Clinical suspicion of an infected aneurysm should be confirmed with laboratory and imaging studies. Laboratory studies should include blood cultures, a complete blood count, and inflammatory markers. It is noted that blood cultures may have poor sensitivity and specificity in the diagnosis of mycotic aneurysm [5]. White cell counts and inflammatory markers are generally, but not always, elevated. Notably, blood cultures can be negative in 25-50 percent of patients and cannot be used to rule out the presence of an infected aneurysm. Initial imaging with a chest radiograph may reveal pulmonary nodules and focal consolidations near the pulmonary vasculature, however, definitive diagnosis, and procedural planning is accomplished with computed tomography angiography (CTA) [1]. CTA allows for timely and effective surgical or endovascular procedural planning [2]. The standard management of infected aneurysms includes antibiotic therapy with surgical debridement and revascularization as needed or endovascular management. Mycotic aneurysms are managed with a prolonged intravenous antibiotic course, similar to infective endocarditis [1]. Broad-spectrum coverage targeting both gram-positive and gram-negative pathogens should be initiated as soon as mycotic aneurysms are suspected and then narrowed pending culture results [1]. Management of pulmonary pseudoaneurysm can include endovascular therapies such as direct coil embolization, endovascular stenting, or embolization of the feeding vessel [1]. Coil embolization is preferred for larger lesions [1]. Surgical management carries a higher risk of morbidity and mortality and involves open thoracotomy, aneurysm resection, and lobectomy of the involved lobes [1]. A high degree of clinical suspicion is required to diagnose pulmonary pseudoaneurysm, given the indolent presentation and rarity of the condition. This is especially true in an atypical patient such as this case, without a history of intravenous drug use or endocarditis. Timely diagnosis is essential to institute appropriate treatment early and prevent nonemergent interventional management.

## 5. Conclusion

Mycotic pulmonary pseudoaneurysm is a rare but life-threatening complication of bacteremia and septic emboli. This is an atypical case of septic embolization from an infected tunneled dialysis catheter. Mycotic pseudoaneurysm should be on the differential of a persistently febrile patient with a known potential source of septic embolization. Early detection is important to instate antibiotic therapy and plan for potential management. In cases with large proximal pulmonary pseudoaneurysms, both procedural and conservative managements carry a high risk of mortality. This case was managed conservatively with subsequent involution of the pulmonary artery pseudoaneurysm.

## Data Availability

The data used to support the findings of this study are included within the article. The patient's information used to compile this case report is not disclosed and is protected under HIPAA.
